# Real-world effectiveness of primary screening with high-risk human papillomavirus testing in the cervical cancer screening programme in China: a nationwide, population-based study

**DOI:** 10.1186/s12916-021-02026-0

**Published:** 2021-07-15

**Authors:** Yanxia Zhao, Heling Bao, Lan Ma, Bo Song, Jiangli Di, Linhong Wang, Yanqiu Gao, Wenhui Ren, Shi Wang, Hai-Jun Wang, Jiuling Wu

**Affiliations:** 1 National Center for Women and Children’s Health, Chinese Center for Disease Control and Prevention, 12 Dahuisi Road, Haidian District, Beijing, 100081 China; 2 Department of Maternal and Child Health, School of Public Health, Peking University, 38 Xueyuan Road, Haidian District, Beijing, 100191 China; 3 National Center for Chronic and Non-communicable Disease Control and Prevention, Chinese Center for Disease Control and Prevention, 27 Nanwei Road, Xicheng District, Beijing, 100050 China

**Keywords:** Uterine cervical neoplasms, Mass screening, Human papillomavirus DNA tests, Cytology

## Abstract

**Background:**

Randomized controlled trials have shown a higher sensitivity and longer negative predictive value of high-risk human papillomavirus (HPV) testing than cytology for cervical cancer screening; however, little is known about the effectiveness of HPV testing in middle-income countries. Understanding the characteristics of HPV testing may increase the priority of HPV testing in health policies. The study aims to evaluate the effectiveness of HPV testing in the national cervical cancer screening programme in China.

**Methods:**

We performed a nationwide, population-based study using individual data from the national cervical cancer screening programme in rural China between 2015 and 2017. The analyses included 1,160,981 women aged 35–64 years who underwent cytology alone or high-risk HPV testing with cytology or genotyping triage. The main outcome was cervical intraepithelial neoplasia 2 or worse (CIN2+). We used multivariate logistic regressions and performed sensitivity analyses with propensity score matching to compare the screening positive, colposcopy referral, detection rate, and positive predictive value (PPV).

**Results:**

The screening positive rates for HPV testing and cytology were 10.1% and 4.0%, respectively. The per protocol colposcopy referral rate of HPV testing was significantly lower than that of cytology (3.5% vs 4.0%), and this difference was mostly due to the low referral threshold of cytology (≥ASC-US). Overall, HPV testing detected more CIN2+ (5.5 vs. 4.4 per 1000, adjusted odds ratio [aOR]=1.18, 95% confidence interval 1.11–1.25) and had a higher PPV (13.8% vs 10.9%, aOR 1.29, 95% CI 1.21–1.37) than cytology. The colposcopy referrals of HPV testing in comparison to cytology differed by income status; it significantly increased in lower-middle-income areas (3.7% vs 3.1%, aOR 1.21, 95% CI 1.17–1.25) and significantly decreased in upper-middle-income areas (3.4% vs 4.9%, aOR 0.69, 95% CI 0.67–0.71). Sensitivity analyses demonstrated the reliability and robustness of the results.

**Conclusions:**

The introduction of HPV testing could improve both the CIN2+ detection rate and efficiency of cervical cancer screening programme, supporting the introduction of primary screening with high-risk HPV testing in China. Further study is needed to investigate the long-term effect of this change.

**Supplementary Information:**

The online version contains supplementary material available at 10.1186/s12916-021-02026-0.

## Background

The World Health Organization (WHO) launched the global elimination plan for cervical cancer through vaccination, high-performance tests, and treatment for precancerous lesions [1]. Randomized controlled trials confirm that human papillomavirus (HPV) testing detects more cervical intraepithelial neoplasia (CIN) and provides greater protection against invasive cervical cancer (ICC) than cytology [2, 3]. However, the increased number of colposcopy referrals in HPV-based screening is still a concern [4, 5]. Limited studies have evaluated the real-world effectiveness of HPV testing in a national-scale cervical cancer screening programme.

Some high-income countries have fully or partially switched from cytology-based screening to HPV-based screening [6], and the introduction of HPV testing in the Netherlands [7] and England [8] has been assessed. These studies compared the effectiveness of HPV testing with cytology triage to cytology with HPV triage, and as expected, both more colposcopy referrals (Netherlands, 3% vs 1%; England, 7% vs 5%) and approximately 1.5 times more CIN2+ detection were found with HPV-based screening than cytology-based screening. More colposcopy referrals may potentially lead to physical or psychological harm [9, 10], and thus, this potential drawback should be considered before the introduction of HPV testing.

An Argentina study yielded similar results as in high-income countries [11], but the evidence regarding the effectiveness of HPV testing and different triages for HPV positive (e.g. cytology triage, HPV genotyping triage, or combined) is still limited in low- and middle-income countries. Therefore, whether to introduce HPV testing is a dilemma for policymakers. Since 2009, China initiated the National Cervical Cancer Screening Programme in Rural Areas (NACCSPRA) and adopted cytology-based screening [12]. However, the morbidity and mortality of cervical cancer remained stable or even increased in the past decade, reflecting the inadequacy of screening coverage and sensitivity [13]. To address the disadvantages of cytology-based screening (e.g. insensitivity, subjectivity, and insufficient cytologists), the programme introduced HPV testing in 2014. This programme provided an opportunity for comparing HPV testing to contemporaneous cytology in the routine screening programme.

This study aimed to assess the effectiveness of HPV testing in the national cervical cancer screening programme and to provide real-world evidence about the introduction of primary screening with HPV testing in China and other similar middle-income countries.

## Methods

### Data source and study design

The NACCSPRA was implemented in more than 1000 counties across China and was used to screen approximately 10 million rural women aged 35–64 years per year [14]. The programme used cytology as the primary screening method, and 17 counties from 10 provinces were selected to monitor the quality of the cytology-based screening. To evaluate HPV testing as primary screening, a large HPV pilot was implemented in 26 provinces. The pilot adopted primary screening with HPV testing, and approximately 520,000 women were screened using HPV testing every year.

This study extracted individual screening data from the programme between January 2015 and December 2017 for a population-based, contemporaneous comparison. We included 131 HPV pilot counties based on the following criteria: (1) had at least 1000 women screened, (2) had more than 70% of colposcopy attendance, and (3) had complete records from one round of screening. Simultaneously, cytological monitoring counties were included as the comparison (Additional file 1: Table S1). The regional distribution of HPV testing or cytology was a consequence of practical conditions and was not subject to randomization. Additionally, women in the study were unvaccinated against HPV because the commercial vaccine was not licensed in mainland China until 2017.

### Cytology-based screening procedures

Women were invited for cytology screening by local physicians. The gynecologist examined the genital tract and cervix with naked eye. Cervical exfoliate cells were obtained by brushes and placed into the medium, and a liquid-based method was used to produce slides. Cytology reading was performed in local hospitals or third-party laboratories based on the Bethesda System. The programme adopted the WHO “screen-and-treat” approach [15], in which women with atypical squamous cells of undetermined significance or worse (ASC-US+) were referred to immediate colposcopy and biopsy if needed (Additional file 1: Fig. S1). Women who had clinically relevant abnormalities (i.e. visible abnormalities with the naked eye or contact bleeding) were directly referred for colposcopy regardless of the results of the primary screening.

### HPV-based screening procedures

Women were invited for cervix examination and HPV-based screening. Clinician-collected specimens were collected, placed into the preservation solution, transported, and stored until processed in the laboratory. The programme required Chinese Food and Drug Administration-approved HPV assays, which detected at least 13 high-risk HPV types (i.e. HPV-16, HPV-18, HPV-31, HPV-33, HPV-35, HPV-39, HPV-45, HPV-51, HPV-52, HPV-56, HPV-58, HPV-59, and HPV-68). Different HPV assays were used depending on the local conditions and we showed the main five types of polymerase chain reaction-based HPV assays (Additional file 1: Table S2).

The pilot adopted two triage strategies for HPV positivity: cytology triage and genotyping triage (Additional file 1: Fig. S2-S3). For cytology triage, reflex cytology was performed on residual HPV-positive samples, and women who were diagnosed as ASC-US+ were referred to colposcopy and biopsy if needed, while those with negative cytology were advised to have intensified screening after 12 months. For genotyping triage, women who were positive for either HPV-16/18 or other HPV with ASC-US+ were referred to colposcopy, while women who were positive for other HPV with negative cytology were advised to have intensified screening after 12 months. Clinically relevant abnormalities were also directly referred. The allocation of triage method depended on the local policy. Due to limited resources and frequency of migration in rural China, the programme did not actively invite women who needed intensified screening but referred approximately 10% of them for opportunistic colposcopy.

### Outcomes

Histological results were classified as negative, CIN1, CIN2/3, or ICC [16]. Adenocarcinoma in situ (AIS) was included in CIN2/3. ICC included adenocarcinoma and adenosquamous carcinoma. We categorized the indicators into three categories to compare HPV testing to cytology: (1) screening positive and colposcopy referral, representing the potential harms of the screening [9, 10]; (2) detection rate for CIN2+ (including CIN2/3 and ICC), representing the benefit; and (3) positive predictive values (PPV), representing the efficiency. Per protocol colposcopy referral was defined as the women who were screened positive and referred to colposcopy according to the protocol. Total referrals were the combination of per protocol referrals and other opportunistic referrals. Overall detection rate of lesions included per protocol detections and other detections in opportunistic colposcopy. PPV was calculated from the histologically defined lesions by the number of women with a positive screen [17]. A positive screen in HPV testing was a test positive with positive HPV-16/18, positive cytology triage, or clinically relevant abnormality.

### Statistical analysis

The main analyses were on a per-protocol basis. We used inverse probability weighting accounting for the screening result and age, to adjust for loss to follow-up in the referral, assuming a similar risk of precancerous lesions or cancer between women who did and did not attend the referral. We also repeated the analyses based on the unweighted data as sensitivity analysis. Given that income status was associated with cervical cancer incidence and the quality of screening [18, 19], we performed stratification analyses by income classifications. We collected the per capita gross domestic product at the county level in 2014 and categorized them into two strata according to the international income classification proposed by the World Bank in 2014 [20]: lower-middle-income areas (US$1046–4085 per year) and upper-middle-income areas (US$4085–12,735 per year).

We tested the differences in the demographic characteristics and indicators with χ^2^. We calculated adjusted odds ratio (aOR) and 95% confidence interval [CI] with multivariable logistic regression for comparisons, adjusting for age, ever screening, and income classification. To balance the confounding factors between the two groups, we conducted sensitivity analyses with propensity score matching. Briefly, we conducted 1:1:1 matching among cytology, HPV testing with cytology triage, and HPV testing with genotyping triage using propensity scores and the calliper matching algorithm with a calliper value of 0.1 standard deviations [21]. All statistical tests were two-sided, and a *P* value less than 0.05 was considered statistically significant. All analyses were conducted with SAS version 9.4 software. This study is reported as per the Strengthening the Reporting of Observational Studies in Epidemiology (STROBE) guidelines (Additional file 2).

## Results

### Characteristics of the study participants

The study included 1,160,981 women, of whom 833,469 underwent HPV testing and 327,512 underwent cytology (Table 1). The two groups were similarly distributed across geographic areas and there were similar numbers of women screened each year from 2015 to 2017 (Additional file 1: Fig. S4). In the HPV group, 243,174 had HPV testing with cytology triage, and 590,295 had HPV testing with genotyping triage. Women in HPV testing were slightly younger than those in cytology (47.2 vs 48.1 years). There was no significant difference in ever screening between the two groups. The proportion of women in the lower-middle-income area was higher among women screened with cytology than among women screened with HPV testing (48.1% vs 29.7%; *P*<.001).

**Table 1:** Characteristics of participants in HPV testing and cytology

	Cytology	HPV testing		
		Overall HPV testing	HPV testing with cytology triage	HPV testing with genotyping triage
**Participants**	327,512	833,469	243,174	590,295
**Mean age** (years [SD])	48.1 (7.6)	47.2 (7.7)	46.6 (7.8)	47.5 (7.6)
**Age group**				
35–44	115,312 (35.2)	327,535 (39.3)	103,752 (42.7)	223,783 (37.9)
45–54	143,483 (43.8)	354,744 (42.6)	98,279 (40.4)	256,465 (43.5)
55–64	68,717 (21.0)	151,190 (18.1)	41,143 (16.9)	110,047 (18.6)
**Ever screening** (yes) ^a^	116,802 (36.2)	284,159 (34.1)	67,754 (27.9)	216,405 (36.7)
**Per protocol** (yes)	324,522 (99.1)	775,379 (93.0)	224,236 (92.2)	551,143 (93.4)
**Place of residence** (lower-middle-income area)	157,394 (48.1)	247,780 (29.7)	43,243 (17.8)	204,537 (34.7)

Note: *HPV* human papillomavirus, *SD* standard deviation

^a^Self-reported ever screening before attendance to the programme

### Relative performance of HPV testing and cytology

**Fig. 1:**
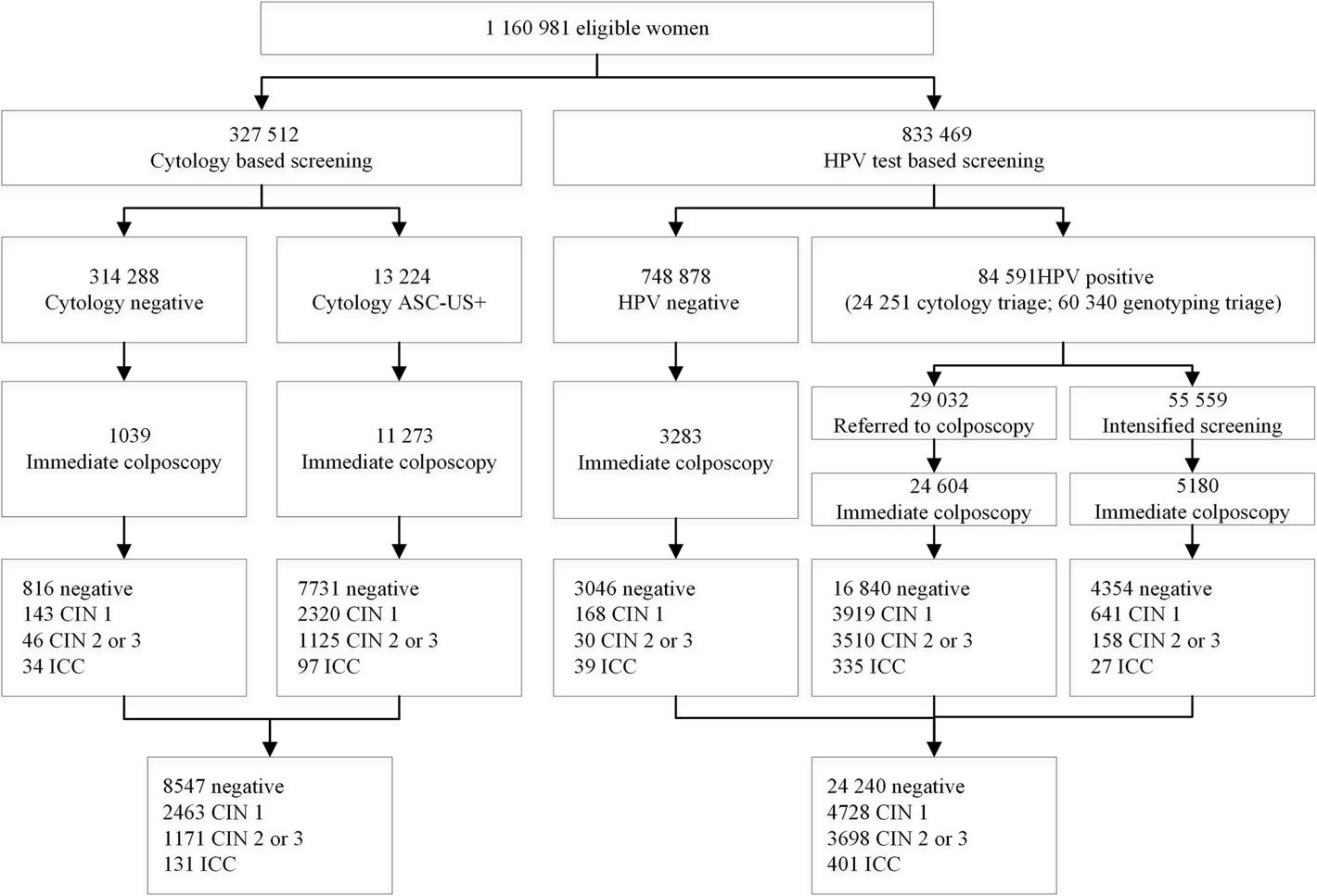
Flow chart of eligible women for HPV testing and cytology in the study. Note: HPV, human papillomavirus; CIN, cervical intraepithelial neoplasia 2 grade or worse; ASC-US, atypical squamous cells of undetermined significance; ICC, invasive cervical cancer

Figure 1 shows the follow-up of the screened women. The proportion of cytological abnormalities was 4.0% (n=13,224), and the proportion of HPV positivity was 10.1% (n=84,591) (*P*<.001). There was no difference in the proportion of HPV positivity between the two HPV subgroups. The women who underwent HPV testing with triage had a lower per protocol colposcopy referral rate than women who underwent cytology alone (3.5% vs 4.0%, *P*<.001). There was no significant difference in the adherence to colposcopy referral between HPV testing and cytology (84.7% vs 85.2%, *P*=0.87). Additionally, 1039 women with normal cytology, 3283 women with HPV negativity, and 5180 women with HPV positivity and negative cytology attended immediate colposcopy.

Table 2 shows the relative performance of HPV testing and cytology. HPV testing detected 3860 CIN2+ (5.5 per 1000) according to the protocol, and cytology detected 1222 CIN2+ (4.4 per 1000). The odds of per protocol CIN2+ detection was higher for HPV testing than for cytology (aOR 1.18, 95% CI 1.11–1.25). The detection rates of both CIN2/3 and ICC for HPV testing were higher than those for cytology (CIN2/3, 5.0 and 4.0 per 1000; ICC, 0.5 and 0.3 per 1000). The PPV for CIN2+ for HPV testing was significantly higher than that for cytology (13.8% vs 10.9%, *P*<.001). The colposcopy referral rates of HPV testing with cytology triage or genotyping triage were 2.2% and 4.0%, respectively. HPV testing with cytology triage had comparable CIN2+ detection with cytology (4.4 vs 4.4 per 1000), but HPV genotyping triage had significantly higher CIN2+ detection than cytology (5.9 per 1000, *P*<.001). The PPV for CIN2+ for both HPV-testing with cytology triage and genotyping triage (16.8% and 13.0%, respectively) was significantly higher than for cytology (*P*<.001 for both).

**Table 2:** Relative effectiveness of HPV testing and cytology

	Cytology	HPV testing					
		Overall HPV testing	aOR (95%CI)	HPV testing with cytology triage	aOR (95%CI)	HPV testing with genotyping triage	aOR (95%CI)
**Screen positivity**, n (%)	13 224 (4.0)	84 591 (10.1)	2.65 (2.59–2.70)	24 251 (10.0)	2.57 (2.52–2.63)	60 340 (10.2)	2.67 (2.62–2.72)
**Per protocol colposcopy referral**, n (%)^a^	13 224 (4.0)	29 032 (3.5)	0.85 (0.83–0.87)	5 256 (2.2)	0.51 (0.49–0.52)	23 776 (4.0)	0.98 (0.96–1.00)
**Overall colposcopy referral**, n (%)	12 312 (4.4)	33 067 (4.5)	1.03 (1.00–1.05)	7 395 (3.3)	0.76 (0.74–0.78)	25 672 (5.0)	1.13 (1.10–1.15)
**Per protocol detection rate of CIN or cancer**, n (per 1000)							
CIN2+	1222 (4.4)	3860 (5.5)	1.18 (1.11–1.25)	962 (4.4)	0.92 (0.85–1.00)	2898 (5.9)	1.28 (1.20–1.36)
CIN2 or 3	1125 (4.0)	3523 (5.0)	1.17 (1.09–1.24)	859 (4.0)	0.89 (0.82–0.97)	2664 (5.4)	1.28 (1.20–1.36)
Invasive cervical cancer	97 (0.3)	337 (0.5)	1.33 (1.08–1.64)	103 (0.5)	1.31 (1.00–1.70)	234 (0.5)	1.34 (1.07–1.66)
**Overall detection rate of CIN or cancer**, n (per 1000) ^b^							
CIN2+	1302 (4.6)	4099 (5.7)	1.17 (1.10–1.24)	1076 (4.9)	0.96 (0.88–1.03)	3023 (6.1)	1.26 (1.18–1.33)
CIN2 or 3	1171 (4.2)	3698 (5.2)	1.17 (1.09–1.24)	945 (4.3)	0.93 (0.85–1.01)	2753 (5.6)	1.27 (1.19–1.35)
Invasive cervical cancer	131 (0.5)	401 (0.6)	1.18 (0.98–1.43)	131 (0.6)	1.23 (0.97–1.56)	270 (0.5)	1.16 (0.95–1.41)
**Positive predictive value**, n (%) ^c^							
CIN2+	1222 (10.9)	4030 (13.8)	1.29 (1.21–1.37)	1045 (16.8)	1.60 (1.47–1.75)	2985 (13.0)	1.22 (1.14–1.30)
CIN2 or 3	1125 (10.0)	3668 (12.6)	1.27 (1.19–1.36)	926 (14.9)	1.52 (1.39–1.66)	2742 (12.0)	1.21 (1.13–1.30)
Invasive cervical cancer	97 (0.9)	362 (1.2)	1.42 (1.16–1.75)	119 (1.9)	2.20 (1.70–2.85)	243 (1.1)	1.24 (0.99–1.54)

Note: *HPV* human papillomavirus, *aOR* adjusted odds ratio, *CI* confidential interval, *CIN* cervical intraepithelial neoplasia, *ICC* invasive cervical cancer, *CI* confidential interval. ^a^ Per protocol colposcopy referral was defined as the women who were screened positive and referred to colposcopy according to the protocol. ^b^ Overall detection rates included cases detected in per protocol colposcopy and others detected in opportunistic colposcopy. ^c^ Positive predictive value represented the detected cases from screened positivity. aOR was calculated by using multivariate logistic regression adjusted for age, ever screening, and income classification

### Relative performance of HPV testing and cytology by income classifications

Table 3 shows that, in lower-middle-income areas, HPV testing had higher per protocol CIN2+ detection and PPV and required more colposcopy referrals than cytology (CIN2+ detection, 4.7 vs 3.0 per 1000; PPV, 12.4% vs 9.9%; colposcopy referral, 3.7% vs 3.1%, *P*<.05 for all). In upper-middle-income areas, the CIN2+ detection for HPV testing was higher than that for cytology without statistical significance (5.7 vs 5.6 per 1000, aOR 1.03, 95% CI 0.96–1.11). Nonetheless, the per protocol colposcopy referral rate was significantly lower for HPV testing than cytology (3.4% vs 4.9%, *P*<.001), and thus the PPV for CIN2+ was higher for HPV testing (14.3% vs 11.4%, *P*<.001).

**Table 3:** Relative effectiveness of HPV testing versus cytology by income classifications

	Lower-middle-income areas			Upper-middle-income areas		
	Cytology	Overall HPV testing	aOR (95%CI)	Cytology	Overall HPV testing	aOR (95%CI)
**Screen positivity** (%, 95%CI)	4879 (3.1)	23 764 (9.6)	3.40 (3.29–3.51)	8345 (4.9)	60 827 (10.4)	2.27 (2.21–2.32)
**Per protocol colposcopy referral** (%, 95%CI)^a^	4879 (3.1)	9050 (3.7)	1.21 (1.17–1.25)	8345 (4.9)	19 982 (3.4)	0.69 (0.67–0.71)
**Overall colposcopy referral** (%, 95%CI)	4206 (2.7)	9721 (3.9)	1.55 (1.50–1.61)	8106 (4.8)	23 346 (4.0)	0.83 (0.80–0.85)
**Per protocol detection rate of CIN or cancer** (per 1000, 95%CI)						
CIN2+	406 (3.0)	993 (4.7)	1.53 (1.37–1.70)	816 (5.6)	2867 (5.7)	1.03 (0.96–1.11)
CIN2 or 3	368 (2.7)	912 (4.3)	1.55 (1.38–1.73)	757 (5.2)	2611 (5.2)	1.01 (0.94–1.09)
Invasive cervical cancer	38 (0.3)	81 (0.4)	1.32 (0.92–1.89)	59 (0.4)	256 (0.5)	1.32 (1.02–1.71)
**Overall detection rate of CIN or cancer** ^b^ (per 1000, 95%CI)						
CIN2+	417 (3.1)	1039 (4.9)	1.56 (1.40–1.73)	885 (6.0)	3060 (6.1)	1.01 (0.95–1.09)
CIN2 or 3	373 (2.8)	938 (4.4)	1.57 (1.41–1.76)	798 (5.5)	2760 (5.5)	1.01 (0.94–1.09)
Invasive cervical cancer	44 (0.3)	101 (0.5)	1.44 (1.03–2.01)	87 (0.6)	300 (0.6)	1.08 (0.86–1.35)
**Positive predictive value** ^c^ (%, 95%CI)						
CIN2+	406 (9.9)	1018 (12.4)	1.23 (1.10–1.38)	816 (11.4)	3012 (14.3)	1.31 (1.21–1.41)
CIN2 or 3	368 (9.0)	936 (11.4)	1.25 (1.11–1.40)	757 (10.6)	2732 (13.0)	1.27 (1.17–1.38)
Invasive cervical cancer	38 (0.9)	82 (1.0)	1.02 (0.71–1.46)	59 (0.8)	280 (1.3)	1.65 (1.27–2.14)

Note: *HPV* human papillomavirus, *aOR* adjusted odds ratio, *CI* confidential interval, *CIN* cervical intraepithelial neoplasia, *ICC* invasive cervical cancer, *CI* confidential interval. ^a^Per protocol colposcopy referral was defined as the women who were screened positive and referred to colposcopy according to the protocol. ^b^ Overall detection rates included cases detected in per protocol colposcopy and others detected in opportunistic colposcopy. ^c^ Positive predictive value represented the detected cases from screened positivity. aOR was calculated by using multivariate logistic regression adjusted for age and ever screening

**Fig. 2:**
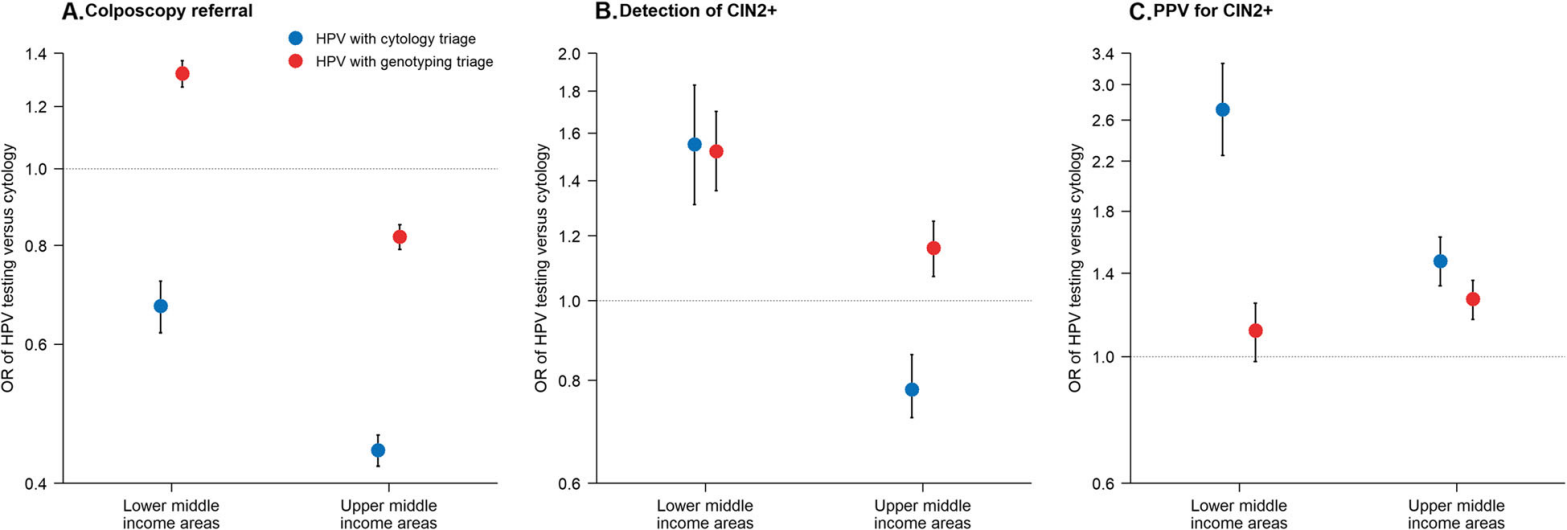
aORs of HPV testing versus cytology alone by income classifications. Note: aOR, adjusted odds ratio; HPV, human papillomavirus; CIN, cervical intraepithelial neoplasia; ICC, invasive cervical cancer; PPV, positive predictive value. Error bars indicated 95% CIs. aOR was calculated by using multivariate logistic regression adjusted for age and ever screening, and the values were conducted by the log transformation on y axis

Figure 2 (Additional file 1: Table S3) compares HPV testing with different triages to cytology alone by income classifications. In lower-middle-income areas, the per protocol colposcopy referral rate was significantly higher for HPV testing with genotyping triage than for cytology (4.0% vs 3.1%, *P*<.001) but lower for HPV testing with cytology triage (2.0% vs 3.1%, *P*<.001). CIN2+ detection for HPV testing with either cytology triage or genotyping triage was significantly higher than for cytology. Finally, an approximately 2.7 times higher PPV for CIN2+ was found for HPV testing with cytology triage compared with cytology, whereas the increase in PPV for HPV testing with genotyping triage was not statistically significant. In upper-middle-income areas, colposcopy referral rates for HPV testing with cytology triage and genotyping triage were 2.2% and 4.0%, respectively, significantly lower than for cytology (*P*<.001 for both). The CIN2+ detection for HPV testing with cytology triage was lower than cytology (4.4 vs 5.6 per 1000, *P*<.001), but for HPV testing with genotyping triage, it was higher than cytology (6.5 vs 5.6 per 1000, *P*<.001). The PPV for CIN2+ was significantly higher than cytology for both HPV testing with cytology triage and genotyping triage (15.7% and 13.9%, respectively, *P*<.001 for both).

### Detection of CIN or invasive cancer in HPV-16/18-positive women

Table 4 shows the detection of lesions in HPV-positive women. In per protocol immediate referrals, the CIN2+ detection rates in women who were HPV-16/18 positive or any HPV positive with cytology ASC-US+ were 17.3% and 24.1%, respectively, significantly higher than that for women who were non-16/18 HPV positive with cytology ASC-US+ (13.8%, *P*<.001). In immediate referrals for women who needed intensified screening, CIN2+ detection rate in women who were any HPV positive with negative cytology was 5.0%, significantly higher than that for women who were positive for non-HPV-16/18 with negative cytology (2.9%, *P*<.001).

**Table 4:** The detection rates of precancerous lesions or cancer in HPV-positive women

	HPV testing with genotyping triage		HPV testing with cytology triage
	HPV-16/18	Non-16-18 HPV genotypes	
**Women who attended immediate colposcopy adherence to the protocol**^a^			
CIN2+, n/N (%)	1922/13 488 (17.3)	902/7167 (13.8)	870/4127 (24.1)
aOR (95% CI)	1.32 (1.22–1.43)	Ref (1.00)	1.96 (1.77–2.16)
Colposcopies per CIN2+ detection	5.8	7.2	4.1
CIN2 or 3, n/N (%)	1736/13 488 (15.6)	861/7167 (13.1)	785/4127 (21.8)
aOR (95% CI)	1.24 (1.14–1.34)	Ref (1.00)	1.82 (1.64–2.01)
ICC, n/N (%)	186/13 488 (1.7)	41/7167 (0.6)	85/4127 (2.4)
aOR (95% CI)	2.75 (2.00–3.78)	Ref (1.00)	3.64 (2.55–5.20)
**Women who needed intensified screening but attended immediate colposcopy** ^b^			
CIN2+, n/N (%)	NA	102/3507 (2.9)	83/1673 (5.0)
aOR (95% CI)	NA	Ref (1.00)	1.92 (1.38–2.67)
Colposcopies per CIN2+ detection	NA	34.5	20.0
CIN2 or 3, n/N (%)	NA	91/3507 (2.6)	67/1673 (4.0)
aOR (95% CI)	NA	Ref (1.00)	1.68 (1.18–2.39)
ICC, n/N (%)	NA	11/3507 (0.3)	16/1673 (1.0)
aOR (95% CI)	NA	Ref (1.00)	3.94 (1.61–9.63)

Note: *HPV* human papillomavirus, *aOR* adjusted odds ratio, *CI* confidential interval, *CIN2+* cervical intraepithelial neoplasia 2 grade or worse, *ICC* invasive cervical cancer, *Ref* reference, *NA* not applicable. ^a^These women included those who were HPV-16 or HPV-18 positive, non-16-18 high-risk HPV positive with abnormal cytology, and any HPV positive with abnormal cytology. ^b^These women included those who were non-16-18 high-risk HPV positive with negative cytology and any HPV positive with negative cytology. aOR was calculated by using multivariate logistic regression adjusted for age, ever screening, and income classification

### Sensitivity analysis

First, the results were consistent between analyses using the weighted and unweighted data (Additional file 1: Table S4). Second, 679,257 women were propensity score matched, and there was no significant difference in the distribution of confounding factors between the two groups (Additional file 1: Table S5). The comparisons in terms of colposcopy referral, CIN2+ detection rate, and PPV between the two groups did not differ from the main results (Additional file 1: Table S6).

## Discussion

### Main findings

This study based on a nationwide cervical cancer screening programme in China found that primary screening with HPV testing had higher CIN2+ detection and PPV than cytology alone in the first round of screening. Unexpectedly, the number of colposcopy referrals after HPV testing was lower than that in cytology. This may be due to the low referral thresholds of cytology-based screening used in China. Moreover, the relative detection and PPV for CIN2+ of HPV testing and cytology were consistent in the lower- and upper-middle-income areas but diverged by different triage strategies. The relative performance of HPV testing and cytology was greater in low-income settings and maybe explained by the lower quality of cytology in rural China.

### Benefits and potential harms of HPV testing

This study showed that HPV testing detected more CIN2+ than cytology, suggesting its benefit in the first round of screening as expected from the results of the previous randomized controlled trials [2, 3, 22, 23]. Furthermore, our study, for the first time, showed that the relative effectiveness of HPV testing versus cytology was consistent in lower- and upper-middle-income areas, supporting the generalizability of its benefit to middle-income areas. The maximally incremental effects of HPV testing compared with cytology were observed in the lower-middle-income areas, with an approximately 50% increase in CIN2+ detection. Increased CIN2+ detection means the opportunity for the immediate treatment. Although the increase of CIN2+ detection in HPV testing was not statistically significant in upper-middle-income areas, the PPV was substantially improved. Previous trials and real-word studies have revealed that the increased detection of CIN2+ in the initial screening reduced the colposcopy referrals and the detection of high-grade lesions in the subsequent round of screening [3, 8, 24].

More screen positives lead to more colposcopy, psychological distress, and overtreatment [9, 10]. The HPV-positive rate in our study was comparable to that in recent population-based studies in China [25] and the Netherlands but higher than those in some European countries [6, 7]. The relatively high HPV prevalence reflected the risk of HPV infection and inadequacy of vaccination and screening in this population. That approximately 2.6 times more positive cases were detected by HPV testing means the high priority of triage of HPV-positive women after introducing HPV-based screening.

The number of colposcopy referrals in HPV testing of our study was comparable with previous studies (2–4%) [6–8, 11], but inconsistent with other studies, the referrals after HPV testing were lower than those after cytology. This should be interpreted with caution. The possible explanations are the low referral threshold of cytology and no follow-up of HPV-positive women with negative cytology. Although many guidelines suggest that women with ASC-US should have HPV triage or intensified screening after 6–12 months, direct colposcopy may be a better choice when HPV testing was not available and good compliance to multiple cytological surveillance is not assured in low-resource settings [26]. As such, China adopted the “screen-and-treat” approach to provide treatment immediately after a positive test, which has been widely adopted by low- and middle-income countries [27, 28]. However, immediate referral for mild cytological dysplasia may be costly and result in overdiagnosis and overtreatment [29]. Our results mean that the introduction of HPV testing could drive more efficient referrals. Nonetheless, the overall referrals (i.e. including those referred per protocol and those referred opportunistically) were higher for HPV testing than for cytology, suggesting the potential increase of referrals for HPV testing after the full course follow-up of all HPV-positive women.

### Factor influencing the introduction of HPV-based screening

Our comparisons of HPV testing with cytology triage or genotyping triage to cytology were discordant in different income settings. This is likely explained by the variations in the quality of cytology-based screening. In lower-middle-income areas, the quality of cytology is generally poor due to insufficient cytologists and inadequate quality assurance, which led to high benefits of HPV testing. However, HPV genotyping triage that needed more referrals may dramatically increase the workload of colposcopy and exacerbated the diagnostic accuracy [30], which potentially contributed to the decrease in PPV. This suggest investigating the affordability of colposcopy before introducing a sensitive HPV strategy in lower-resource settings. In upper-middle-income areas, the quality of cytology is relatively high, with a high positive rate and PPV. This yielded a significantly lower CIN2+ detection in HPV-testing with cytology triage. Loss-to-follow-up of HPV-positive women with normal cytology also reduces the detection of lesions. To detect more lesions, a more sensitive strategy, such as HPV genotyping strategy, and a full course follow-up after HPV-positive results are needed. Ultimately, policy makers should consider setting-specific factors that may affect screening strategies before introducing the HPV testing.

The introduction of HPV testing has to reconsider the follow-up of women with HPV positivity with negative cytology. Multiple visits to complete a full screening would be difficult to implement in low-resource settings and decrease the effectiveness [27]. Our study indicated that HPV genotyping triage provided better risk stratification and required fewer women to attend a close testing. This would reduce the number of loss-to-follow-up for triage testing and lose fewer cases with CIN2+ than cytology triage. However, the advantage of HPV-16/18 triage may be temporary because HPV vaccination would expand soon after the availability of domestic HPV vaccine [31]. As such, the follow-up of women who need intensified screening should be enhanced and further studies need to assess the cost-effectiveness of new triage approaches in postvaccination screening, such as DNA methylation [32], or p16-INK4A overexpression [33].

### Strengths and limitations of this study

This study, to our knowledge, was the largest study thus far that conducted a comprehensive assessment of primary screening with HPV testing in a middle-income country. Its large sample size enabled robust comparisons of HPV-based and cytology-based screening strategies by income classifications. Particularly, the participants in this study were all unvaccinated, and the conclusions would be applicable for countries where expanded vaccination programme have not yet been carried out.

Several limitations should be discussed. First, the study did not randomly allocate the screening methods. However, the risk of precancerous or cancerous lesions would be comparable between the two groups because of the similar geographic distribution, an assumption supported by the sensitivity analyses. Second, the absolute difference between the two groups should be interpreted prudently. CIN2+ detection and colposcopy referral would be underestimated for HPV testing due to no follow-up for HPV positivity with negative cytology, and the loss-to-follow-up in the immediate referral also affects the estimation. Although the extent of the impact was unknown, we believe this would attenuate the benefits of HPV testing but not reverse the conclusions, apart from colposcopy referrals. Third, CIN3+ is a preferable endpoint but this programme did not require the separation of CIN2/3 because of the immediate treatment for CIN2+. Finally, there were different HPV assays and devices in the study; however, all assays have been well verified clinically and had high agreement.

Our study has substantial implications for the prevention of cervical cancer in middle-income countries, particularly for countries that currently use cytology with a low referral threshold. Although the cost of HPV testing may delay full accessibility in low-resource setting, a modelling study showed that early introducing HPV testing would have the opportunity to prevent more cases of cervical cancer and save more quality-adjusted life years [34]. Changing the cytology threshold to a high cutoff value and prolonging the follow-up frame would provide more ways to evaluate HPV-based screening and optimize the programme for postvaccination screening.

## Conclusions

Our study found that primary screening with HPV testing improved the effectiveness of the national cervical cancer screening programme. Our results support the introduction of HPV testing in China and similar middle-income countries and has implications for the global elimination of cervical cancer. Long-term follow-up data are needed to further investigate the protective efficacy of HPV testing in reducing the incidence of cervical cancer.

## Supplementary Information

**Additional file 1: Table S1**. The distribution of eligible women screened by HPV testing or cytology. **Table S2.** Overview of the HPV assays specifications and related parameters in the programme. **Table S3.** ORs of HPV testing with different triages versus cytology alone by income classifications. **Table S4.** Sensitivity analyses for relative effectiveness of HPV testing and cytology based on unweighted data. **Table S5.** Demographic characteristics of women in pre-propensity score matching and post-propensity score matching. **Table S6.** Sensitivity analysis with propensity score matching for comparisons of HPV testing and cytology alone. **Fig. S1** Flow diagram of primary cytology screening. **Fig. S2** Flow diagram of primary HPV testing with cytology triage. **Fig. S3** Flow diagram of primary HPV testing with partially genotyping triage. **Fig. S4** Screened women by geographic areas and calendar, China 2015-17.)

**Additional File 2.** STROBE checklist for the reports of observational studies.

## Data Availability

The data that support the findings of this study are available from the corresponding author, upon reasonable request.

## Abbreviations

aOR: Adjusted odds ratio
ASC-US: Atypical squamous cells of undetermined significance
CI: Confidence interval
CIN: Cervical intraepithelial neoplasia
HPV: Human papillomavirus
ICC: Invasive cervical cancer
NACCSPRA: National Cervical Cancer Screening Programme in Rural Areas
WHO: World Health Organization
