# Supplementary material for: Real-world effectiveness of primary screening with high-risk human papillomavirus testing in the cervical cancer screening programme in China: a nationwide, population-based study
Source: BMC Med. 2021 Jul 15;19:164. doi: 10.1186/s12916-021-02026-0 (PMC8281674; doi:10.1186/s12916-021-02026-0)
Supplement: Supplementary file 1 — Additional file 1: Table S1. The distribution of eligible women screened by HPV testing or cytology. Table S2. Overview of the HPV assays specifications and related parameters in the programme. Table S3. ORs of HPV testing with different triages versus cytology alone by income classifications. Table S4. Sensitivity analyses for relative effectiveness of HPV testing and cytology based on unweighted data. Table S5. Demographic characteristics of women in pre-propensity score matching and post-propensity score matching. Table S6. Sensitivity analysis with propensity score matching for comparisons of HPV testing and cytology alone. Fig. S1 Flow diagram of primary cytology screening. Fig. S2 Flow diagram of primary HPV testing with cytology triage. Fig. S3 Flow diagram of primary HPV testing with partially genotyping triage. Fig. S4 Screened women by geographic areas and calendar, China 2015-17.) [file 12916_2021_2026_MOESM1_ESM.docx]

**Additional File 1**

**Supplement to:** **Real-world effectiveness of** **primary screening with high-risk human papillomavirus testing** **in the cervical cancer screening programme in China: a nationwide, population-based study.**

**Author:** Yanxia Zhao, Heling Bao, Lan Ma, Bo Song, Jiangli Di, Linhong Wang, Yanqiu Gao, Wenhui Ren, Shi Wang, Hai-Jun Wang, Jiuling Wu.

[Table S1. The distribution of eligible women screened by HPV testing or cytology. 2](#_Toc73651718)

[Table S2. Overview of the HPV assays specifications and related parameters in the programme. 3](#_Toc73651719)

[Table S3. ORs of HPV testing with different triages versus cytology alone by income classifications. 4](#_Toc73651720)

[Table S4. Sensitivity analyses for relative effectiveness of HPV testing and cytology based on unweighted data. 5](#_Toc73651721)

[Table S5. Demographic characteristics of women in pre-propensity score matching and post-propensity score matching. 6](#_Toc73651722)

[Table S6. Sensitivity analysis with propensity score matching for comparisons of HPV testing and cytology alone. 7](#_Toc73651723)

[Fig. S1 Flow diagram of primary cytology screening. 8](#_Toc73651724)

[Fig. S2 Flow diagram of primary HPV testing with cytology triage. 9](#_Toc73651725)

[Fig. S3 Flow diagram of primary HPV testing with partially genotyping triage. 10](#_Toc73651726)

[Fig. S4 Screened women by geographic areas and calendar, China 2015-17. 11](#_Toc73651727)

## Table S1. The distribution of eligible women screened by HPV testing or cytology.

| **Geographic areas** | **Provinces** | **HDI index**  **in 2014** | **Cytology** | | **HPV testing** | |
| --- | --- | --- | --- | --- | --- | --- |
|  |  |  | **Number of counties** | **Number of screened women** | **Number of counties** | **Number of screened women** |
| Eastern China | Beijing | 0.887 | NA | NA | 5 | 49 545 |
|  | Zhejiang | 0.798 | NA | NA | 6 | 57 017 |
|  | Guangdong | 0.784 | 2 | 61 918 | 3 | 31 054 |
|  | Shandong | 0.769 | NA | NA | 4 | 29 970 |
|  | Fujian | 0.758 | 1 | 6441 | 20 | 57 998 |
|  | Hainan | 0.738 | NA | NA | 5 | 48 254 |
|  | Hebei | 0.735 | 2 | 29 832 | 3 | 21000 |
| Central China | Hubei | 0.755 | 2 | 24 953 | 4 | 61 074 |
|  | Shanxi | 0.738 | NA | NA | 21 | 38 429 |
|  | Hunan | 0.735 | 2 | 45 432 | 4 | 56 806 |
|  | Jiangxi | 0.726 | NA | NA | 7 | 33 996 |
|  | Anhui | 0.720 | NA | NA | 9 | 45 290 |
| Western China | Inner Mongolia | 0.766 | 1 | 3953 | 8 | 52 654 |
|  | Shannxi | 0.751 | 1 | 36 718 | 3 | 56 883 |
|  | Chongqing | 0.747 | NA | NA | 4 | 60 534 |
|  | Ningxia | 0.727 | NA | NA | 3 | 5002 |
|  | Sichuan | 0.720 | NA | NA | 5 | 55 215 |
|  | Xinjiang | 0.718 | NA | NA | 9 | 32 434 |
|  | Guangxi | 0.713 | NA | NA | 4 | 39 467 |
|  | Qinghai | 0.694 | NA | NA | 4 | 35 114 |
|  | Gansu | 0.689 | NA | NA | 1 | 8017 |
|  | Guizhou | 0.673 | 2 | 63 684 | 4 | 50 045 |
|  | Yunnan | 0.668 | 2 | 47 022 | 4 | 55 127 |
| Northeast China | Liaoning | 0.798 | 2 | 18 900 | 2 | 29 794 |
|  | Jilin | 0.768 | NA | NA | 2 | 24 515 |
|  | Heilongjiang | 0.755 | NA | NA | 5 | 49 804 |

Note: Data are presented as number. HPV=human papillomavirus. NA= not available.

## Table S2. Overview of the HPV assays specifications and related parameters in the programme.

| **HPV assay Abbr.** | Roche | Hybribio Corp | ZJ Bio-Tech | SanSure | YanengBIO |
| --- | --- | --- | --- | --- | --- |
| **HPV assay** | Cobas polymerase chain reaction-based platform | polymerase chain reaction-based platform | polymerase chain reaction-based platform | polymerase chain reaction-based platform | polymerase chain reaction-based platform |
| **DNA amplifier and hybridization oven** | Cobas Z 480 | PE Applied Biosystem GeneAmp PCR System 9600  GeneAMP PCR System 9700 | SLAN real‐time thermo‐cycler  Roche LightCycler 480  LineGene9600 Series | Stratagene Mx3000P  SLAN-96P  Roche Light | Eppendorf Mastercycler Gradient, Germary;  MJ PTC-100, USA;  ABI9700, USA  FinePCR Combi-H12, Korea; FYY-3, China |
| **PCR volume** | 125 μL | 24 μL | 36 μL | 45 μL | 15 μL |
| **DNA volume** | 25 μL | 1 μL | 4 μL | 5 μL | 5 μL |
| **High-risk HPV genotypes detected** | HPV/16/18/31/33/35,‐39/45/51/52/56/58/59/66/68 | HPV16/18/31/33/35/39/45/51/52/53/56/58/59/66/68 | HPV16/18/31/33/35/39/45/51/52/56/58/59/66/68/82 | HPV16/18/31/33/35/39/45/51/52/53/56/58/59/66/68 | HPV16/18/31/33/35/3945/51/52/53/56/58/59/66/68 |
| **Detection technology** | Real time fluorescence PCR | PCR-reverse dot blot hybridization | Real time fluorescence PCR | Real time fluorescence one-step fast release techology | PCR-reverse dot blot |
| **Internal quality control** | human housekeeping gene β-globin was used as an endogenous internal control | human housekeeping gene β-globin was used as an endogenous internal control | human housekeeping gene β-globin was used as an endogenous internal control | UNG premier andβ-globin for process validity of individual sample and input sufficiency | β-globin for process validity of individual sample and input sufficiency |
| **Within-group agreement (agreement rate and kappa for overall HPV)** | NA | Cobas: 95.71%; 0.914 | Cobas: 95.24%; 0.905  YanengBio: 97.7%; kappa 0.953 | Cobas: 94.76%; 0.895 | Cobas: 95.72%; 0.839 (0.794-0.884) |

Reference:

1. Feng QH, Wang Y, Bao KY, Zuo P, Li MZ, Xie YJ, Zhao C, et al. Comparison an analysis of domestic human papillomavirus genotyping tests and Cobas 4800 HPV test for cervical cancer screening. Prog Obstet Gynecol, 2020, 29 (7): 499-04

2. Zhang L, Dai Y, Chen J, Hong L, Liu Y, Ke Q, Chen Y, Cai C, Liu X, Chen Z. Comparison of the performance in detection of HPV infections between the high-risk HPV genotyping real time PCR and the PCR-reverse dot blot assays. J Med Virol. 2018 Jan;90(1):177-183.

3. Xue P, Gao LL, Yin J, Han LL, Zhao J, Li L, Seery S, Han XY, Li TY, Jiang Y, Chen W, Shen J. A direct comparison of four high-risk human papillomavirus tests versus the cobas test: Detecting CIN2+ in low-resource settings. J Med Virol. 2019 Jul;91(7):1342-1350.

## Table S3. ORs of HPV testing with different triages versus cytology alone by income classifications.

|  | **Lower-middle-income areas** | | | | **Upper-middle-income areas** | | | |
| --- | --- | --- | --- | --- | --- | --- | --- | --- |
|  | **HPV testing with**  **cytology triage** | **aOR**  **(95% CI)** | **HPV testing with**  **genotyping triage** | **aOR**  **(95% CI)** | **HPV testing with**  **cytology triage** | **aOR**  **(95% CI)** | **HPV testing with**  **genotyping triage** | **aOR**  **(95% CI)** |
| **Screened positivity** | 3912 (9.0) | 3.24 (3.10−3.39) | 19 852 (9.7) | 3.43 (3.32−3.54) | 20 399 (10.2) | 2.23 (2.17−2.29) | 40 488 (10.5) | 2.28 (2.23−2.34) |
| **Per protocol colposcopy referral** | 882 (2.0) | 0.67 (0.62−0.72) | 8168 (4.0) | 1.32 (1.27−1.37) | 4374 (2.2) | 0.44 (0.42−0.45) | 15 608 (4.0) | 0.82 (0.79−0.84) |
| **Overall colposcopy referral** | 923 (1.9) | 0.76 (0.70−0.82) | 8898 (4.4) | 1.72 (1.65−1.78) | 6572 (3.3) | 0.68 (0.66−0.70) | 16 774(4.3) | 0.90 (0.88−0.93) |
| **Per protocol detection rate of CIN or cancer** | |  |  |  |  |  |  |  |
| CIN2+ | 185 (4.8) | 1.55 (1.31−1.83) | 808 (4.7) | 1.52 (1.36−1.70) | 777 (4.4) | 0.78 (0.72−0.86) | 2090 (6.5) | 1.16 (1.07−1.25) |
| CIN2 or 3 | 167 (4.3) | 1.57 (1.32−1.87) | 745 (4.4) | 1.55 (1.38−1.74) | 692 (3.9) | 0.75 (0.68−0.82) | 1919 (6.0) | 1.14 (1.06−1.24) |
| Invasive cervical cancer | 18 (0.5) | 1.35 (0.77−2.36) | 63 (0.4) | 1.32 (0.91−1.91) | 85 (0.5) | 1.29 (0.94−1.75) | 171 (0.5) | 1.33 (1.02−1.75) |
| **Overall detection rate of CIN or cancer** ^†^ | |  |  |  |  |  |  |  |
| CIN2+ | 190 (4.9) | 1.56 (1.32−1.83) | 849 (4.9) | 1.56 (1.40−1.74) | 886 (4.9) | 0.82 (0.75−0.89) | 2174 (6.7) | 1.12 (1.04−1.20) |
| CIN2 or 3 | 171 (4.4) | 1.59 (1.34−1.88) | 767 (4.5) | 1.57 (1.40−1.76) | 774 (4.3) | 0.79 (0.72−0.86) | 1986 (6.1) | 1.12 (1.04−1.21) |
| Invasive cervical cancer | 19 (0.5) | 1.28 (0.75−2.21) | 82 (0.5) | 1.47 (1.04−2.07) | 112 (0.6) | 1.16 (0.89−1.51) | 188 (0.6) | 1.04 (0.82−1.32) |
| **Positive predictive value** ^⁋^ | |  |  |  |  |  |  |  |
| CIN2+ | 187 (24.4) | 2.71 (2.25−3.26) | 831 (11.2) | 1.11 (0.98−1.24) | 858 (15.7) | 1.47 (1.33−1.62) | 2154 (13.9) | 1.26 (1.16−1.36) |
| CIN2 or 3 | 169 (22.1) | 2.72 (2.25−3.29) | 767 (10.4) | 1.12 (0.99−1.27) | 757 (13.9) | 1.37 (1.24−1.52) | 1975 (12.7) | 1.24 (1.14−1.34) |
| Invasive cervical cancer | 18 (2.4) | 1.91 (1.08−3.38) | 64 (0.9) | 0.92 (0.63−1.34) | 101 (1.8) | 2.40 (1.77−3.24) | 179 (1.2) | 1.42 (1.08−1.87) |

Note: HPV=human papillomavirus. aOR=adjusted odd ratio. CI=confidential interval. CIN2+=cervical intraepithelial neoplasia 2 grade or worse. ICC=invasive cervical cancer. CI=confidential interval. ^†^ Overall detection rates were calculated from all detected cases divided by all women according to the intent-to-screen approach. ^⁋^ Positive predictive value was calculated from detected cases divided by screened positive women according to protocol at initial screening. aOR was calculated by using multivariate logistic regression adjusted for age and ever screening.

## Table S4. Sensitivity analyses for relative effectiveness of HPV testing and cytology based on unweighted data.

|  | Cytology | HPV testing | | | | | |
| --- | --- | --- | --- | --- | --- | --- | --- |
|  |  | Overall HPV testing | aOR (95%CI) | HPV testing with cytology triage | aOR (95%CI) | HPV testing with genotyping triage | aOR (95%CI) |
| **Per protocol detection rate of CIN or cancer**, n (per 1000) | | |  |  |  |  |  |
| CIN2+ | 1222 (3.7) | 3860 (4.6) | 1.17 (1.09−1.25) | 962 (4.0) | 0.96 (0.88−1.05) | 2898 (4.9) | 1.25 (1.18−1.35) |
| CIN2 or 3 | 1125 (3.4) | 3523 (4.2) | 1.16 (1.08−1.24) | 859 (3.5) | 0.93 (0.85−1.01) | 2664 (4.5) | 1.25 (1.17−1.34) |
| Invasive cervical cancer | 97 (0.3) | 337 (0.4) | 1.33 (1.06−1.67) | 103 (0.4) | 1.36 (1.02−1.81) | 234 (0.4) | 1.32 (1.04−1.67) |
| **Overall detection rate of CIN or cancer**, n (per 1000) ^*^ | |  |  |  |  |  |  |
| CIN2+ | 1302 (4.0) | 4099 (4.9) | 1.16 (1.09−1.24) | 1076 (4.4) | 0.99 (0.92−1.08) | 3023 (5.2) | 1.23 (1.15−1.31) |
| CIN2 or 3 | 1171 (3.6) | 3698 (4.5) | 1.16 (1.09−1.24) | 945 (3.9) | 0.97 (0.89−1.06) | 2753 (4.7) | 1.24 (1.16−1.33) |
| Invasive cervical cancer | 131 (0.4) | 401 (0.5) | 1.16 (0.95−1.42) | 131 (0.4) | 1.26 (0.98−1.62) | 270 (0.5) | 1.12 (0.91−1.39) |
| **Positive predictive value**, n (%) ^†^ | |  |  |  |  |  |  |
| CIN2+ | 1222 (9.2) | 4030 (11.3) | 1.27 (1.18−1.35) | 1045 (13.9) | 1.57 (1.44−1.72) | 2985 (10.6) | 1.19 (1.11−1.28) |
| CIN2 or 3 | 1125 (8.5) | 3668 (10.3) | 1.24 (1.16−1.34) | 926 (12.4) | 1.49 (1.36−1.64) | 2742 (9.8) | 1.18 (1.10−1.27) |
| Invasive cervical cancer | 97 (0.7) | 362 (1.0) | 1.41 (1.12−1.76) | 119 (1.5) | 2.18 (1.65−2.87) | 243 (0.8) | 1.21 (0.96−1.54) |

Note: HPV=human papillomavirus. aOR=adjusted odd ratio. CI=confidential interval. CIN=cervical intraepithelial neoplasia. ICC=invasive cervical cancer. CI=confidential interval. The estimations were not weighted. * Overall detection rates were calculated according to the intent-to-screen approach. † Positive predictive value represented the detected cases from screened positivity according to protocol in the initial screening and those who should have intensified screening. aOR was calculated by using multivariate logistic regression adjusted for age, ever screening, and income classification.

## Table S5. Demographic characteristics of women in pre-propensity score matching and post-propensity score matching.

|  | **Cytology** | **HPV testing** | ***P* value** |
| --- | --- | --- | --- |
| **Pre-matching** |  |  |  |
| Participants, (n, %) | 327 512 (28.6) | 833 469 (71.4) |  |
| Age (years, [median, IQR]) | 48.0 (42.0, 53.0) | 47.0 (41.0, 53.0) | <0.001 |
| Age group (n, %) |  |  | <0.001 |
| 35-44 | 115 312 (35.2%) | 327 535 (39.3%) |  |
| 45-54 | 143 483 (43.8%) | 354 744 (42.6%) |  |
| 55-64 | 68 717 (21.0%) | 151 190 (18.1%) |  |
| Ever screening^⁎^ (n, %) |  |  | 0.63 |
| Yes | 116 802 (36.2%) | 284 159 (34.1%) |  |
| No | 208 591 (63.8%) | 548 376 (65.9%) |  |
| **Post-matching** |  |  |  |
| Participants, (n, %) | 226 419 (33.3) | 452 838 (66.7) |  |
| Age (years, [median, IQR]) | 47.0 (42.0, 53.0) | 47.0 (41.0, 53.0) | 0.14 |
| Age group (n, %) |  |  | 0.26 |
| 35-44 | 82 687 (36.5%) | 176 700 (39.0%) |  |
| 45-54 | 98 089 (43.3%) | 193 082 (42.6%) |  |
| 55-64 | 45 643 (20.2%) | 83 056 (18.3%) |  |
| Ever screening^⁎^ (n, %) |  |  | 0.84 |
| Yes | 70 147 (30.9%) | 135 029 (29.8%) |  |
| No | 156 272 (69.1%) | 317 809 (70.2%) |  |

Note: IQR=inter-quartile range. Data are presented as number (%) or median (inner-quartile range). HPV=human papillomavirus. ^⁎^ Yes: self-reported ever screened for cervical cancer before the current screening. No: self-reported never attended cervical cancer screening. ^†^ p value for χ² test.

## Table S6. Sensitivity analysis with propensity score matching for comparisons of HPV testing and cytology alone.

|  | **aOR of overall HPV testing versus cytology alone (95% CI) ^†^** | ***P* value** | **aOR of HPV testing with cytology triage versus cytology alone (95% CI) ^†^** | ***P* value** | **aOR of HPV testing with genotyping triage versus cytology alone (95% CI) ^†^** | ***P* value** |
| --- | --- | --- | --- | --- | --- | --- |
| **Screened positivity** | 2.77 (2.71−2.84) | <0.001 | 2.70 (2.63−2.78) | <0.001 | 2.84 (2.77−2.91) | <0.001 |
| **Per protocol colposcopy referral** | 0.79 (0.77−0.81) | <0.001 | 0.53 (0.51−0.55) | <0.001 | 1.04 (1.01−1.07) | 0.01 |
| **Overall colposcopy referral** | 1.02 (0.99−1.05) | 0.19 | 0.81 (0.78−0.84) | <0.001 | 1.22 (1.18−1.26) | <0.001 |
| **Per protocol detection of CIN2 or worse** | | | | |  |  |
| CIN2+ | 1.15 (1.06−1.25) | 0.001 | 1.01 (0.92−1.11) | 0.85 | 1.28 (1.16−1.40) | <0.001 |
| CIN2 or 3 | 1.12 (1.03−1.22) | 0.01 | 0.97 (0.88−1.08) | 0.97 | 1.26 (1.15−1.39) | <0.001 |
| ICC | 1.46 (1.09−1.95) | 0.01 | 1.49 (1.08−2.06) | 0.02 | 1.44 (1.05−1.99) | 0.03 |
| **Overall detection rate of CIN or cancer** | |  |  |  |  |  |
| CIN2+ | 1.13 (1.04−1.22) | 0.003 | 1.03 (0.94−1.13) | 0.59 | 1.22 (1.12−1.34) | <0.001 |
| CIN2 or 3 | 1.12 (1.03−1.22) | 0.007 | 1.00 (0.91−1.11) | 0.95 | 1.24 (1.13−1.36) | <0.001 |
| Invasive cervical cancer | 1.16 (0.91−1.48) | 0.23 | 1.22 (0.92−1.60) | 0.16 | 1.11 (0.84−1.46) | 0.47 |
| **Positive predictive value**, n/N (%) | |  |  |  |  |  |
| CIN2+ | 1.32 (1.21−1.44) | <0.001 | 1.68 (1.52−1.86) | <0.001 | 1.13 (1.02−1.24) | 0.02 |
| CIN2 or 3 | 1.28 (1.17−1.40) | <0.001 | 1.59 (1.43−1.77) | <0.001 | 1.11 (1.01−1.22) | 0.04 |
| ICC | 1.67 (1.26−2.22) | <0.001 | 2.40 (1.74−3.29) | <0.001 | 1.26 (0.92−1.73) | 0.16 |

Note: aOR=adjusted odd ratio. CI=confidential interval. CIN2+=cervical intraepithelial neoplasia 2 grade or worse. ICC=invasive cervical cancer. 679 257 women were matched by 1:1:1 with propensity score for sensitivity analysis, with 226 419 women in cytology group, HPV test triaged by cytology group, and HPV test triaged by partial genotyping group. ^†^aOR was calculated by using multivariable logistic regression adjusted for age, ever screening, and income classifications.

**
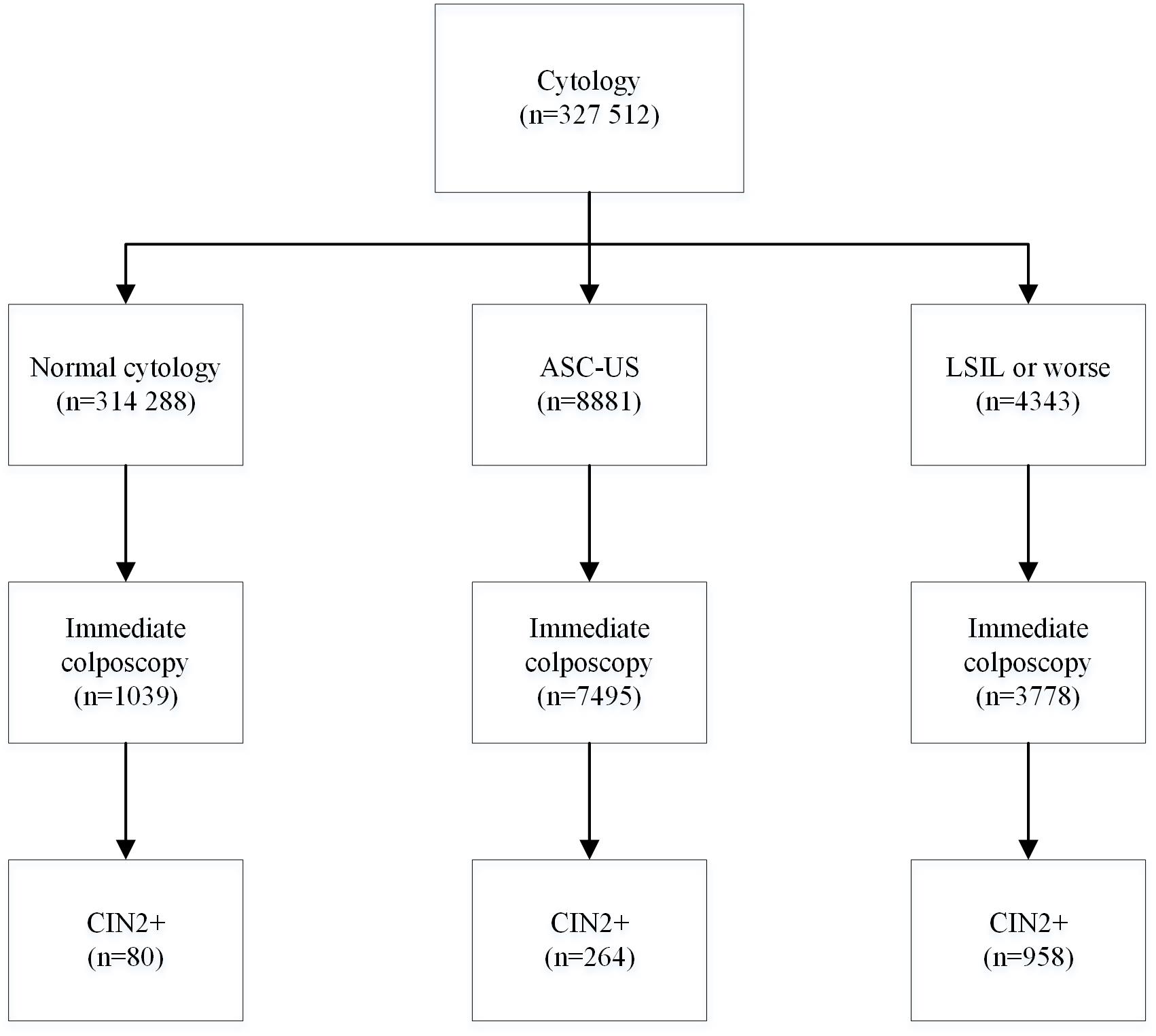
**

## Fig. S1 Flow diagram of primary cytology screening.

Abbreviations: ASC-US=atypical squamous cells of undetermined significance; LSIL=low-grade squamous intraepithelial lesion; CIN2+=cervical intraepithelial neoplasia 2 grade or worse.


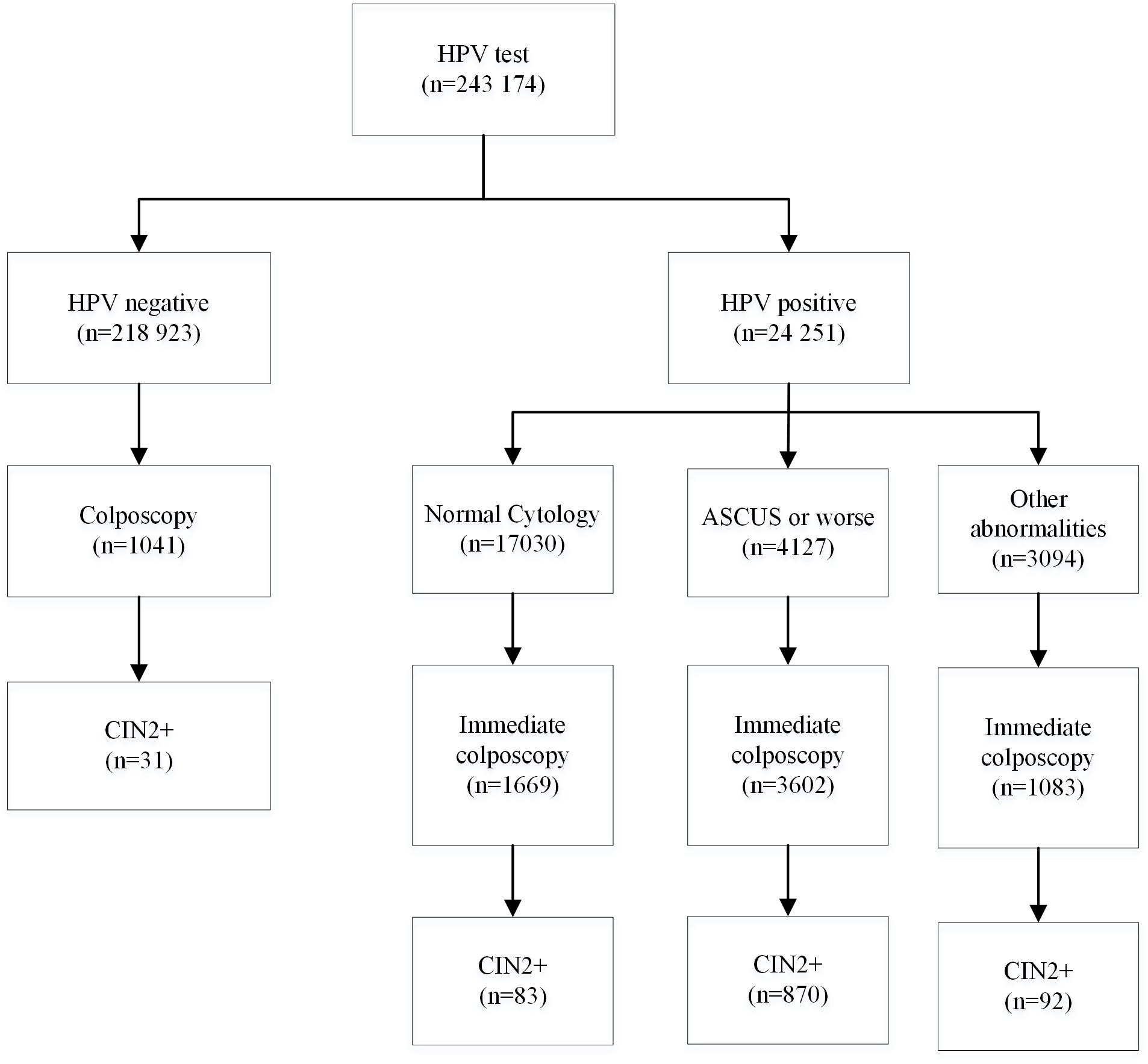


## Fig. S2 Flow diagram of primary HPV testing with cytology triage.

Abbreviations: HPV=human papillomavirus; ASC-US=atypical squamous cells of undetermined significance; CIN2+=cervical intraepithelial neoplasia 2 grade or worse.

Other abnormalities represented women who had normal cytology but clinically relevant abnormalities (such as visible abnormalities with naked eyes or contact bleeding).


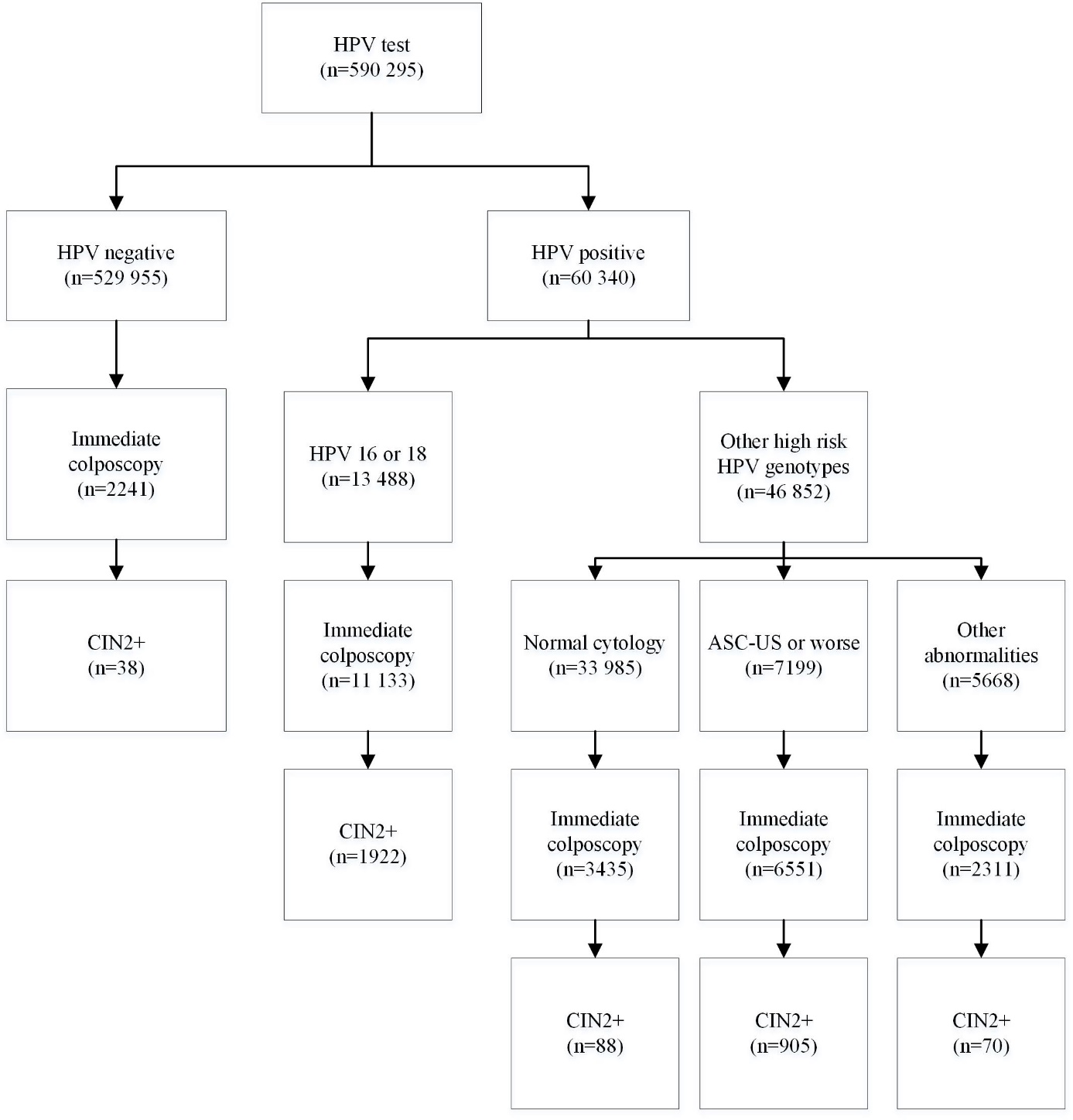


## Fig. S3 Flow diagram of primary HPV testing with partially genotyping triage.

Abbreviations: HPV=human papillomavirus; ASC-US=atypical squamous cells of undetermined significance; CIN2+=cervical intraepithelial neoplasia 2 grade or worse.

Other abnormalities represented women who had normal cytology but clinically relevant abnormalities (such as visible abnormalities with naked eyes or contact bleeding).


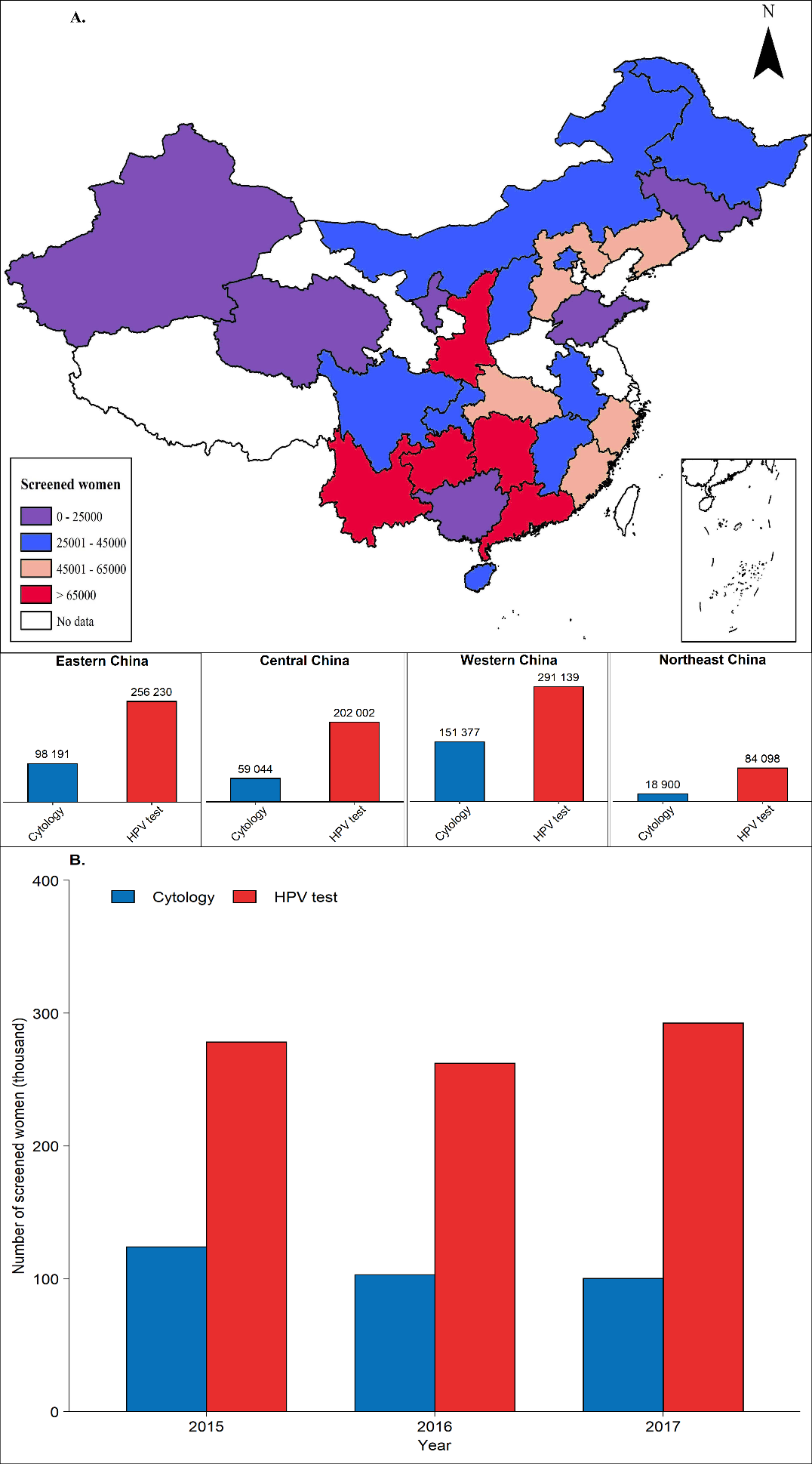


## Fig. S4 Screened women by geographic areas and calendar, China 2015-17.

Note: HPV=human papillomavirus. The 31 provinces in China were categorized as four regions, eastern China, central China, western China, and northeast China.
