# Supplementary material for: Real-world effectiveness of primary screening with high-risk human papillomavirus testing in the cervical cancer screening programme in China: a nationwide, population-based study
Source: BMC Med. 2021 Jul 15;19:164. doi: 10.1186/s12916-021-02026-0 (PMC8281674; doi:10.1186/s12916-021-02026-0)
Supplement: Supplementary file 2 — Additional File 2. STROBE checklist for the reports of observational studies. [file 12916_2021_2026_MOESM2_ESM.docx]

**Additional File 2**

**Supplement to:** **Real-world effectiveness of** **primary screening with high-risk human papillomavirus testing** **in the cervical cancer screening programme in China: a nationwide, population-based study.**

**Author:** Yanxia Zhao, Heling Bao, Lan Ma, Bo Song, Jiangli Di, Linhong Wang, Yanqiu Gao, Wenhui Ren, Shi Wang, Hai-Jun Wang, Jiuling Wu.

**S1** **Checklist.** STROBE checklist for the reports of observational studies

|  | Item No | Recommendation | Reported on page |
| --- | --- | --- | --- |
| **Title and abstract** | 1 | (*a*) Indicate the study’s design with a commonly used term in the title or the abstract | Abstract |
|  |  | (*b*) Provide in the abstract an informative and balanced summary of what was done and what was found | Abstract |
| Introduction | | |  |
| Background/rationale | 2 | Explain the scientific background and rationale for the investigation being reported | Page 5 |
| Objectives | 3 | State specific objectives, including any prespecified hypotheses | Page 6 |
| Methods | | |  |
| Study design | 4 | Present key elements of study design early in the paper | Page 6-8  Fig. S1-S3 |
| Setting | 5 | Describe the setting, locations, and relevant dates, including periods of recruitment, exposure, follow-up, and data collection | Page 6-8  Fig.S4, Table S1 S2 |
| Participants | 6 | (*a*) Give the eligibility criteria, and the sources and methods of selection of participants | Page 6-7 |
| Variables | 7 | Clearly define all outcomes, exposures, predictors, potential confounders, and effect modifiers. Give diagnostic criteria, if applicable | Page 9 |
| Data sources/ measurement | 8* | For each variable of interest, give sources of data and details of methods of assessment (measurement). Describe comparability of assessment methods if there is more than one group | Page 9 |
| Bias | 9 | Describe any efforts to address potential sources of bias | Page 9-10 |
| Study size | 10 | Explain how the study size was arrived at | NA |
| Quantitative variables | 11 | Explain how quantitative variables were handled in the analyses. If applicable, describe which groupings were chosen and why | Page 9-10 |
| Statistical methods | 12 | (*a*) Describe all statistical methods, including those used to control for confounding | Page 10 |
|  |  | (*b*) Describe any methods used to examine subgroups and interactions | Page 9-10 |
|  |  | (*c*) Explain how missing data were addressed | Page 9 |
|  |  | (*d*) If applicable, describe analytical methods taking account of sampling strategy | NA |
|  |  | (*e*) Describe any sensitivity analyses | Page 9-10 |
| Results | | |  |
| Participants | 13* | (a) Report numbers of individuals at each stage of study—eg numbers potentially eligible, examined for eligibility, confirmed eligible, included in the study, completing follow-up, and analysed | Page 10 Table 1 |
|  |  | (b) Give reasons for non-participation at each stage | Page 10, Table 1 |
|  |  | (c) Consider use of a flow diagram | Page 11, Fig. 1 |
| Descriptive data | 14* | (a) Give characteristics of study participants (eg demographic, clinical, social) and information on exposures and potential confounders | Page 10, Table 1 |
|  |  | (b) Indicate number of participants with missing data for each variable of interest | NA |
| Outcome data | 15* | Report numbers of outcome events or summary measures | Page 11  Table 2 |
| Main results | 16 | (*a*) Give unadjusted estimates and, if applicable, confounder-adjusted estimates and their precision (eg, 95% confidence interval). Make clear which confounders were adjusted for and why they were included | Page 11-13  Table 2, Table 3, Table 4 |
|  |  | (*b*) Report category boundaries when continuous variables were categorized | Page 12 |
|  |  | (*c*) If relevant, consider translating estimates of relative risk into absolute risk for a meaningful time period | NA |
| Other analyses | 17 | Report other analyses done—eg analyses of subgroups and interactions, and sensitivity analyses | Page 12-14,  Fig. 2  Table S4− S6 |
| Discussion | | |  |
| Key results | 18 | Summarise key results with reference to study objectives | Page 14 |
| Limitations | 19 | Discuss limitations of the study, taking into account sources of potential bias or imprecision. Discuss both direction and magnitude of any potential bias | Page 18 |
| Interpretation | 20 | Give a cautious overall interpretation of results considering objectives, limitations, multiplicity of analyses, results from similar studies, and other relevant evidence | Page 14-17 |
| Generalisability | 21 | Discuss the generalisability (external validity) of the study results | Page 18 |
| Other information | | |  |
| Funding | 22 | Give the source of funding and the role of the funders for the present study and, if applicable, for the original study on which the present article is based | Page 20 |
